# Correction to “Effectiveness, safety and overall satisfaction of early postpartum placement of hormonal IUD compared with standard procedure: An open‐label, randomized, multicenter study”

**DOI:** 10.1111/aogs.14563

**Published:** 2023-04-07

Lichtenstein Liljeblad K, Kopp Kallner H, Brynhildsen J. Effectiveness, safety and overall satisfaction of early postpartum placement of hormonal IUD compared with standard procedure: an open‐label, randomized, multicenter study. *Acta Obstet Gynecol Scand*. 2022;101:424–430.

The text in result section under the abstract heading and on page 427, right hand column, first two paragraphs are incorrect. The corrected text is given below.

**Corrected text:**

**Abstract**

**Results**

The study was prematurely stopped according to the protocol due to an expulsion rate > 20% in the early group. No pregnancies occurred. Fifty‐two women were randomized to early and 49 women to standard insertion. In the early group, 23/52 (44.2%) of the intrauterine devices were expelled. After expulsion, 10 women chose to have another hormonal intrauterine device placed but still fewer women (37/52, 71%) in the early group used the hormonal intrauterine device method at study completion. No expulsions occurred in the standard group, but 6/49 Accurate: 6/49 (12.2%) requested removal and 41/49 (83.7%, *p* = 0.13) had used the hormonal intrauterine device method continuously for 1 year.

**On page 427, in the Results section right column the corrections to the text are as follows:**

In the early group there were two removals (2/52, 3.8%) on patient request due to perceived side effects (mood changes and itching). In the standard group there were six removals 6/49 (12.2%) on patient request, in two cases due to pregnancy intention and four removals due to perceived side effect (daily spottings, frequent spottings, disturbed libido and mood changes). There were two perforations (2/49, 4.1%) in the standard group, both diagnosed more than a year after application and after completion of the study. One was a partial perforation diagnosed due to pregnancy intention, and one was a complete perforation diagnosed during a check‐up due to unintended early pregnancy.

A total of 10 women in the early group chose to have a new hormonal IUD placed after the first one was partially or completely expelled. Thus, 37/52 (71%) of the women in the early group had used the hormonal intrauterine device method continuously at study completion compared with 41/49 (83.7%, *p* = 0.13) in the standard group. In the early group one woman chose to have an implant inserted, resulting in use of long‐acting reversible contraception in 38/52 women (73.1%) at the closure of the study. In the standard group, one woman chose to have an implant inserted and one chose to have a Cu‐IUD placed after removal of the hormonal IUD, resulting in continued use of long‐acting reversible contraception in 43/49 (87.8%, *p* = 0.064) women at study closure 1 year after IUD placement.

We apologize for this error.

